# Integrated effects of Kampo treatment on gastrointestinal symptoms and stress in patients with functional dyspepsia: a preliminary prospective observational study

**DOI:** 10.3389/fphar.2025.1685656

**Published:** 2025-11-21

**Authors:** Lian Liang, Hongyang Li, Hirokazu Doi, Yaxuan Jiang, Satoshi Tashiro, Jiying Sun, Akihiro Kawahara, Shiro Oka, Masanori Ito, Keiko Ogawa-Ochiai

**Affiliations:** 1 Department of Kampo Clinical Center, Hiroshima University Hospital, Hiroshima, Japan; 2 Department of Information and Management Systems, Nagaoka University of Technology, Niigata, Japan; 3 Department of Cellular Biology, Research Institute for Radiation Biology and Medicine, Hiroshima University, Hiroshima, Japan; 4 Department of Gastroenterology, Graduate School of Biomedical and Health Sciences, Hiroshima University, Hiroshima, Japan; 5 Department of General Internal Medicine, Hiroshima University Hospital, Hiroshima, Japan

**Keywords:** traditional Japanese medicine, Kampo formulas, Kampo, functional dyspepsia, dyspepsia, stress

## Abstract

**Aim:**

This preliminary, single-center prospective observational study aimed to investigate the effects of Kampo treatment on improving gastrointestinal symptoms and reducing stress levels in patients with Functional Dyspepsia (FD).

**Methods:**

Adult patients diagnosed with FD were included and their background characteristics were collected using a Fundamental State Questionnaire. The Gastrointestinal Symptom Rating Scale (GSRS) was used to assess gastrointestinal symptom improvement, and the Profile of Mood States Second Edition-Adult Short Form (POMS-2A) was used to evaluate stress relief both before and after treatment. Paired t-tests were used to compare the GSRS and POMS-2A scores before and after treatment. One-way ANOVA was applied to explore whether there were differences in efficacy among the three Kampo formulas, while Multiple linear regression was used to analyze the associations between patient characteristics and treatment outcomes.

**Results:**

Forty-one patients with FD were included, and thirty-three were included in the final analysis (N = 41, n = 33 analyzed). Following an average of 52 days of Kampo treatment (bukuryoin, heiisan, rikkunshito), there was a significant improvement in gastrointestinal symptoms, such as hunger, pain, constipation, and gastrectasia, as well as overall symptoms. Additionally, there was a significant decrease in negative mood components (anger and depression) and total mood disturbance. Marital status, smoking habits and sleep quality may serve as significant factors influencing the outcomes of Kampo treatment.

**Conclusion:**

In this exploratory and preliminary study, following Kampo treatment (bukuryoin, heiisan, rikkunshito), the trend of improvements in gastrointestinal symptoms and stress levels were observed in patients with FD. Additionally, marital status, smoking habits and sleep quality may serve as significant factors influencing treatment outcomes.

## 1 Introduction

Functional Dyspepsia (FD) is widely acknowledged as one of the most prevalent gastrointestinal disorders. The worldwide prevalence of FD ranges from 11% to 30% (Mahadeva and Goh, 2006). FD is characterized by chronic symptoms originating in the gastroduodenal region, without any underlying systemic, organic, or metabolic disease (Tack et al., 2006). FD significantly impacts health-related quality of life across the physical, psychological, and social domains. Economic development has accelerated the pace of life, accompanied by numerous pressures and negative emotions. Studies suggest an intricate connection between FD and anxiety as well as depression (Carpinelli et al., 2023). Both basic research and clinical studies have provided insights into the complex pathogenesis of FD, which includes gastric sensorimotor dysfunction and psychiatric instability (Carbone and Tack, 2014).

Pharmacological treatments, such as *Helicobacter pylori* eradication, H_2_ receptor antagonists, proton pump inhibitors, prokinetics, and antidepressants, have been developed to address FD symptoms (Lacy et al., 2012). However, their effectiveness remains limited, as they do not fully address the complex and heterogeneous pathophysiology of FD. Despite active drug use, many patients experience persistent symptoms, reflecting the need for more holistic and individualized treatment approaches (Miwa et al., 2022).

Kampo formulas (traditional Japanese medicinal formulas) provide a holistic and safe approach to healthcare and are commonly used in the clinical management of FD in Japan. The effectiveness and safety of commonly used Kampo formulas for alleviating FD has been demonstrated (Tan et al., 2020). Enteric nervous system, known as the “second brain,” operates independently via the enteric nervous system, influencing digestion, mood, and health through the gut-brain axis (Cheng et al., 2024). There is a robust correlation between FD and psychological factors (Jiang et al., 2015). The prescriptions of Kampo formulas are guided by the principle of “harmony between mind and body,” targeting both physical and mental symptoms simultaneously (Mayer et al., 2022). However, few studies have substantiated the effectiveness of Kampo treatment in alleviating specific symptoms and relieving stress conditions of FD.

Rikkunshito and heiisan derived from Prescriptions of the Bureau of Taiping People’s Welfare Pharmacy during the Song Dynasty. They are widely used to alleviate gastrointestinal symptoms and improve upper gastrointestinal symptoms in patients with FD. A study showed that rikkunshito significantly reduced dyspepsia and alleviated epigastric pain and postprandial fullness (Suzuki et al., 2014). A previous randomized controlled trial also showed that rikkunshito may offer simultaneous relief from both gastrointestinal and psychological symptoms in FD patients (Tominaga et al., 2018). Heiisan improved gastrointestinal motor function in FD rats by decreasing the leptin concentration in serum and the brain-gut axis, and by increasing the CCK concentration in gastrointestinal tissue (Liang et al., 2018). In addition, a pharmacodynamic study showed that heiisan significantly improved anti-fatigue capacity in mice (Zhang et al., 2016). Bukuryoin, a classical Kampo formula documented in the Synopsis of Prescriptions of the Golden Chamber, has traditionally been used to treat phlegm-fluid retention and associated gastrointestinal disorders. Previous studies have indicated that Wai Tai Fuling Yin, a related formulation, is primarily utilized in the treatment of digestive diseases, with notable efficacy in enhancing gastrointestinal function (Jeffri, 2024). However, the underlying mechanisms of bukuryoin remain poorly understood. These prior experiments indicate, rikkunshito, bukuryoin, and heiisan possibly by enhancing gastrointestinal motility and modulating the brain-gut axis and neuroendocrine responses, thereby contributing to both gastrointestinal and stress-related symptom relief.

Rikkunshito efficacy has been validated by randomized controlled trials (Tominaga et al., 2018) — yet two other formulas, bukuryoin and heiisan, lack sufficient empirical evidence supporting their efficacy. Additionally, in clinical settings, physicians prescribe Kampo formulas based on individualized syndrome differentiation and treatment rather than fixed indications, meaning these three formulas are frequently applied interchangeably according to patient-specific syndromes. Given the clinical relevance of these formulas and the existing evidence gaps, this study included rikkunshito, bukuryoin, and heiisan as the target interventions.

Nevertheless, there remains a lack of empirical evidence assessing the integrated effects of Kampo on both digestive symptoms and psychological stress using standardized clinical measures. Therefore, this preliminary, single-center, prospective observational study aimed to explore the extent to which Kampo formulas are associated with improvements in gastrointestinal symptoms and reductions in stress-related mood disturbances in patients with FD, while accounting for lifestyle and demographic factors that may influence treatment response.

## 2 Materials and methods

### 2.1 Medication

The compositions of the Kampo formulas (rikkunshito, bukuryoin, nichinto, heiisan, or anchusan) used in this study are listed in Table 1. All Kampo formulas used in this study were manufactured by Tsumura and Co. (Tokyo, Japan) and approved for ethical use by The Ministry of Health, Labour and Welfare (2021). Their manufacturing methods, components, and quantities comply with the standards of the Japanese Pharmacopoeia (The Ministry of Health, Labour and Welfare, 2021). The Japanese Pharmacopoeia is the official compendium issued by the MHLW, which defines the origins and characteristics of listed botanical drugs and Kampo formulas, and specifies their permissible limits, purity tests, and analytical methods to ensure quality, safety, and consistency. Detailed information on each formula can be accessed through the STORK database (http://.mpdb.nibiohn.go.jp/stork/), managed by the National Institutes of Biomedical Innovation, Health and Nutrition (NIBN). Although this was an observational study, a brief *a priori* safety review was performed based on pharmacopoeial and post-marketing information to ensure appropriate monitoring of known risks. The Kampo formulas used in this study are prescription medicines that can only be provided to patients with a doctor’s prescription. These medicines are covered under Japan’s National Health Insurance system.

**TABLE 1:** The components of Kampo Formulas for patients.

Crude drugs	Scientific name	Bukuryoin	Rikkunshito	Heiisan	Nichinto	Anchusan
Poria sclerotium	Wolfiporia cocos ryvarden et gilbertson [polyporaceae; poria sclerotium]	5.0 g	4.0 g		5.0 g	
Atractylodes lancea rhizome	Atractylodes lancea de candolle; atractylodes chinensis koidzumi [asteraceae; atractylodes lancea rhizome]	4.0 g	4.0 g	4.0 g		
Citrus unshiu peel	Citrus unshiu markowicz; citrus reticulata blanco [rutaceae; citri unshiu pericarpium]	3.0 g	2.0 g	3.0 g	4.0 g	
Ginseng	Panax ginseng C. A. Meyer [araliaceae; ginseng radix et rhizoma]	3.0 g	4.0 g			
Immature orange	Citrus aurantium linné var. daidai Makino, Citrus aurantium linné; citrus natsudaidai hayata [rutaceae; aurantii fructus immaturus]	1.5 g				
Ginger	Zingiber officinale roscoe [zingiberaceae; zingiberis rhizoma]	1.0 g	0.5 g	0.5 g	1.0 g	
Pinellia tuber	Pinellia ternata breitenbach [araceae; pinellia tuber]		4.0 g		5.0 g	
Jujube	Ziziphus jujuba miller var. inermis rehder [rhamnaceae; ziziphi fructus]		2.0 g	2.0 g		
Glycyrrhiza	Glycyrrhiza uralensis fischer; glycyrrhiza glabra linné [fabaceae; glycyrrhizae radix et rhizoma]		1.0 g	1.0 g	1.0 g	1.0 g
Magnolia bark	Magnolia obovata thunb.; magnolia officinalis rehder et E. H. Wilson; magnolia officinalis var. biloba rehder et E. H. Wilson [magnoliaceae; magnoliae cortex]			3.0 g		
Cinnamon bark	Cinnamomum cassia J. Presl [lauraceae; cinnamomi cortex]					4.0 g
Corydalis tuber	Corydalis turtschaninovii besser forma yanhusuo Y. H. Chou et C. C. Hsu [papaveraceae; corydalis tuber]					3.0 g
Oyster shell	Ostrea gigas thunberg [ostreidae; oyster shell]					3.0 g
Fennel	Foeniculum vulgare miller [apiaceae; foeniculi fructus]					1.5 g
Amomum seed	Amomum villosum loureiro var. xanthioides T. L. Wu et S. J. Chen,amomum villosum loureiro var. Villosum; amomum longiligulare T. L. Wu [zingiberaceae; amomi fructus]					1.0 g
Alpinia officinarum rhizome	Alpinia officinarum hance [zingiberaceae; alpiniae officinarum rhizoma]					0.5 g

### 2.2 Patients

This study was designed as a preliminary prospective observational study conducted at Hiroshima University Hospital from 2 September 2021, to 27 December 2023. No formal sample size calculation was performed because this was an exploratory study. Based on clinical feasibility and the number of eligible patients with FD treated annually at Hiroshima University Hospital, a target of approximately 40 participants was set. Meanwhile, similar prospective studies (Gerson and Triadafilopoulos, 2005) on FD typically enroll 40 participants. We recruited adult patients diagnosed with FD based on Rome IV criteria (Aziz et al., 2020), who had been prescribed one of the following Kampo formulations as part of their standard clinical care: rikkunshito, bukuryoin, nichinto, heiisan, or anchusan, for an average of 52 days. The exclusion criteria were as follows: pregnancy, possibility of pregnancy, a history of abdominal surgery, diabetes mellitus, celiac disease or inflammatory bowel disease, active psychiatric conditions; current participation in another interventional clinical study; and inability to provide informed consent or complete study procedures. This study was approved by the Ethical Committee for Epidemiology of Hiroshima University (Approval No. E2021-2483). Prior to enrollment, all participants were fully informed of the study procedures and potential risks, and written informed consent was obtained in accordance with the Declaration of Helsinki.

The Kampo formulas were prescribed according to routine clinical practice, with no modifications to dosage or duration for the purpose of this study. Patients were managed under the supervision of experienced Kampo physicians. The post-treatment assessment was conducted immediately after the end of treatment. Clinical data were collected in an observational manner, without influencing therapeutic decision-making.

### 2.3 Outcomes

Primary outcomes are changes in Gastrointestinal Symptom Rating Scale (GSRS). The secondary outcomes are changes in the Profile of Mood States Second Edition-Adult Short Form (POMS-2A) and the influence of lifestyle and demographic factors on treatment response in FD patients.

#### 2.3.1 Fundamental State Questionnaire survey

Before Kampo treatment, patients with FD completed the Fundamental State Questionnaire to gather background information, including marital status, occupation, sleep quality, appetite, smoking, drinking, Kampo formulas, and symptoms such as cold feet and stiff shoulders.

#### 2.3.2 Gastrointestinal symptom rating scale (GSRS)

The GSRS is a validated questionnaire (Tominaga et al., 2007; Jindal et al., 2024; Wardenaar et al., 2025) used to assess gastrointestinal (GI) symptoms. The patients completed the procedure before and after the Kampo treatment. It includes 16 items, including stomachache, heartburn, acid reflux, hunger pains, nausea, rumbling, gastrectasia, burp, fart, constipation, diarrhea, loose stools, hard stools, urgent need for defecation, incomplete evacuation feeling and total, scored on a 7-point Likert scale (1–7) to evaluate symptom frequency and severity over the past week. Higher scores indicated more severe GI symptoms.

#### 2.3.3 Profile of mood states second edition-adult short form (POMS 2-A)

The POMS 2-A questionnaire (Trask et al., 2001; Li et al., 2023) was used to assess the stress-relief effects of the Kampo treatment. Patients completed the POMS 2-A questionnaire during these two phases, enabling the measurement of changes in negative mood (including anger, confusion, depression, fatigue, and tension), positive mood (vigor and friendliness), and overall mood disturbance (TMD). Higher scores indicated a greater severity of mood symptoms reported by patients with FD.

### 2.4 Statistical analyses

Statistical analyses were performed using SPSS version 28, which was categorized into three main components and visualized with Prism 8 for OS X (Version 8.4.3). Initially, the effectiveness of the Kampo treatment was assessed by comparing the POMS 2-A and GSRS scores before and after treatment using paired t-tests. We also calculated the responder rate, defined as the proportion of patients who showed continuous improvement in self-reported symptom scores. The second component examined whether there are differences in efficacy among the three Kampo formulas, which were tested using one-way ANOVA. The third component involved multiple linear regression analyses to examine associations between patient characteristics and treatment outcomes—changes in GSRS and POMS 2-A scores, both calculated as pre-treatment minus post-treatment values. For GSRS, the independent variables included age, BMI, gender (female/male), occupation (employed/unemployed), marital status (married/spinsterhood), sleep quality (good/bad), appetite (good/bad), smoking (yes/no), drinking habits (yes/no), and specific Kampo formulas (bukuryoin, rikkunshito, heiisan). For POMS 2-A, age and gender were excluded because they were already adjusted in the scoring. Included only patients with complete data and excluded those with missing covariates. To account for multiple comparisons, p-values were adjusted using the Benjamini-Hochberg procedure to control the false discovery rate (FDR). Both unadjusted and FDR-adjusted p-values (q-values) were reported to ensure transparency and maintain statistical rigor. Significant effects of background characteristics were further investigated using independent-sample t-tests. Statistical significance was defined as p < 0.05.

## 3 Results

### 3.1 Safety results

Adverse events (AEs) and adverse drug reactions (ADRs) were assessed within the observation period for FD. No serious or non-serious AEs/ADRs occurred, and no participants withdrew due to these events. No clinically significant laboratory abnormalities were observed.

### 3.2 Fundamental State Questionnaire survey

**FIGURE 1:**
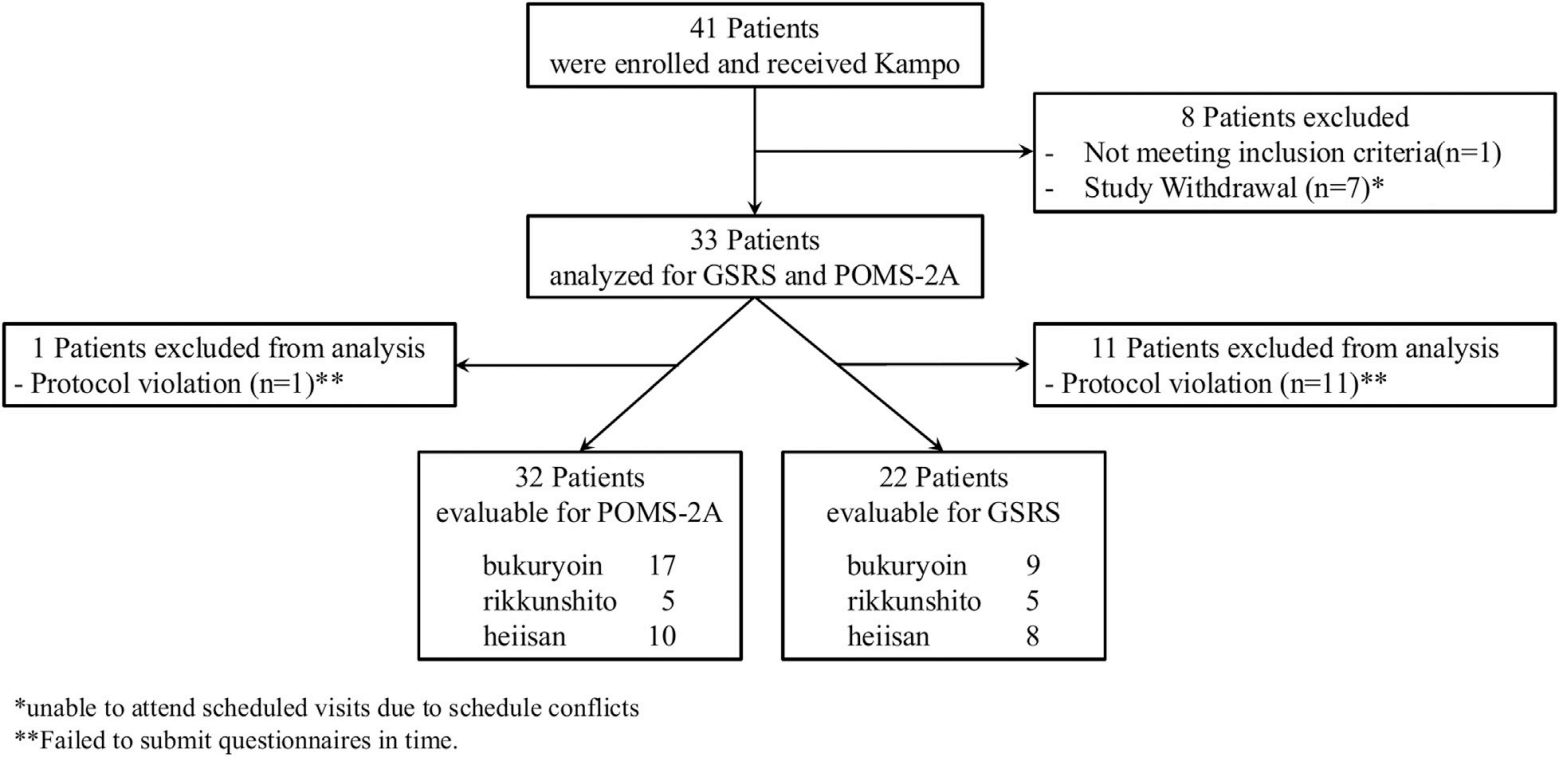
Patient disposition per Consolidated Standards of Reporting Trials guidelines.

Among the 41 patients enrolled, 33 completed the study and were included in the final analysis. The patient disposition is illustrated in Figure 1. Patients received Kampo formulas for an average of 52 days, administered 2–3 times daily before meals. Table 2; Supplementary Table S1 presents the patients’ demographic characteristics. Among the five Kampo formulas specified in the eligibility criteria, only rikkunshito, bukuryoin, and heiisan were prescribed to enrolled patients. One-way ANOVA and Multiple linear regression analysis showed no significant differences in efficacy among the Kampo formulas (Supplementary Table S2-S5). The prescription features were as follows: bukuryoin for cold feet, stiff shoulders, and tired eyes; rikkunshito for frequent urination, cold sensitivity, and cold hands; and heiisan for lumbago and cold feet (Table 3).

**TABLE 2:** Patient demographic characteristics.

Items	Number	Mean ± SD
Age	33	53.88 ± 20.25
Height (cm)	33	161.82 ± 8.63
Weight (kg)	33	54.67 ± 11.13
BMI	33	20.79 ± 3.52

**TABLE 3:** Result of fundamental state questionnaire survey.

Characteristics	Bukuryoin	Rikkunshito	Heiisan
Cold feet	13 (68%)	3 (43%)	9 (82%)
Stiff shoulders	13 (68%)	4 (57%)	4 (36%)
Tired eyes	16 (84%)	5 (71%)	9 (82%)
Experiencing frequent urination	9 (47%)	5 (71%)	8 (73%)
Sensitivity to cold	13 (68%)	6 (86%)	8 (73%)
Cold hands	6 (32%)	4 (57%)	5 (45%)
Lumbago	10 (53%)	2 (29%)	8 (73%)
Feel like there is something in their throat	6 (32%)	1 (14%)	3 (27%)

### 3.3 Gastrointestinal symptom rating scale (GSRS)

**FIGURE 2:**
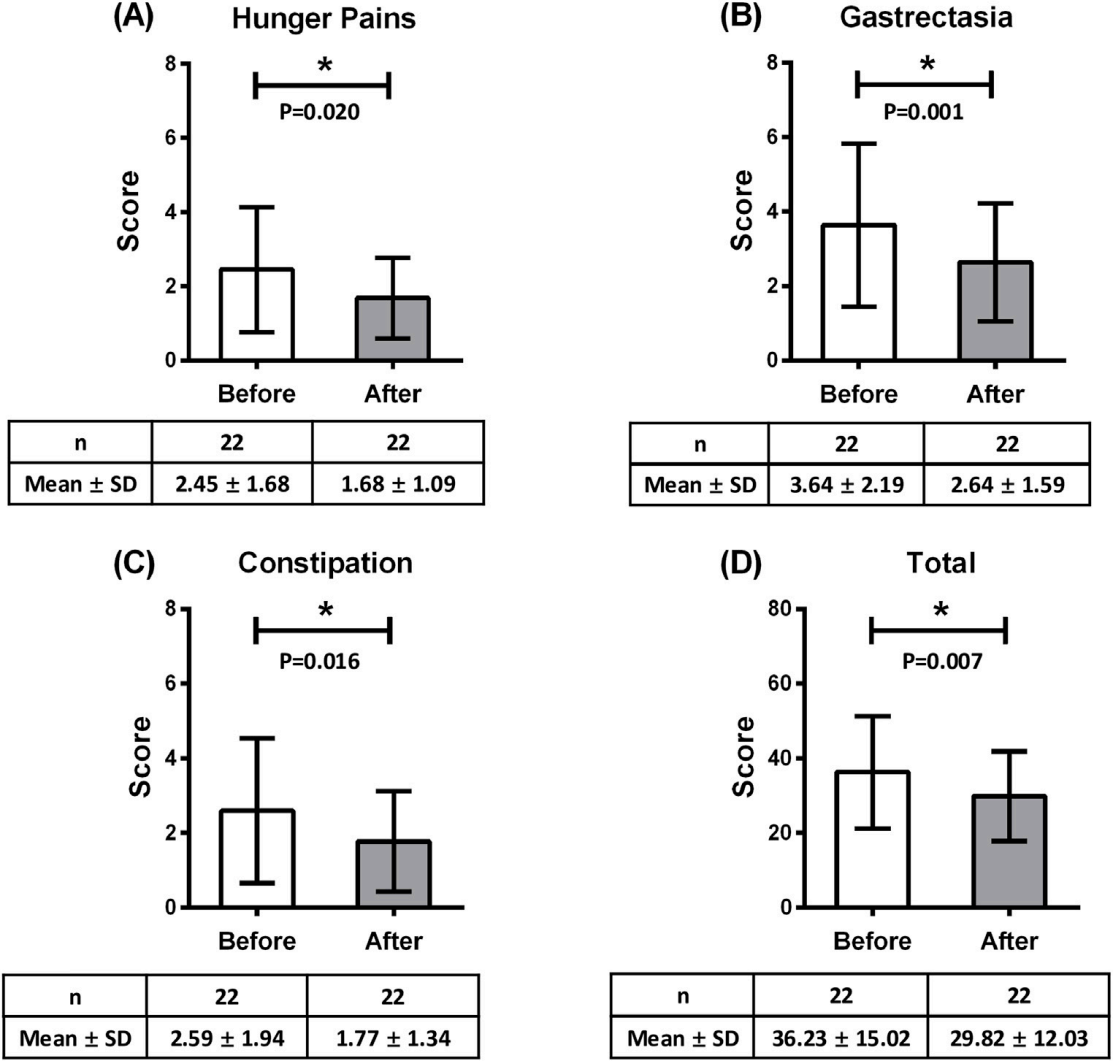
Kampo treatment–induced changes in **(A)** hunger pains, **(B)** gastrectasia, **(C)** constipation, and **(D)** total gastrointestinal symptoms. *p < 0.05.

We conducted a paired t-test to compare the GSRS scores before and after Kampo treatment (Table 4; Supplementary Table S6). Significant reductions were observed in hunger pains, gastrectasia, constipation and total scores before and after Kampo treatment. Hunger pains decreased by 0.77 points (95% CI [-1.010, −0.059]; Cohen’s d = −0.53, p = 0.020, q = 0.033). Gastrectasia decreased by 1.00 points (95% CI [-1.296, −0.276]; Cohen’s d = −0.79, p = 0.001, q = 0.005). Constipation decreased by 0.82 points (95% CI [-1.035, −0.079]; Cohen’s d = -0.56, p = 0.016, q = 0.112). Total scores decreased by 6.41 points (95% CI [-1.126, −0.150]; Cohen’s d = −0.64, p = 0.007, q = 0.018) (Figure 2). The overall responder rate for gastrointestinal symptoms under Kampo treatment was 63.6% (95% CI [40.9–81.8]). Among the specific Kampo formulas, the responder rate of bukuryoin was 77.8% (95% CI [44.4–100.0]), that of heiisan was 62.5% (95% CI [25.0–87.5]), and that of rikkunshito was 40.0% (95% CI [0.0–80.0]).

**TABLE 4:** Paired t-test results of the Gastrointestinal Symptom Rating Scale (GSRS).

GSRS items	Mean ± SD		Cohen’s d	95% CI		t	P value
	Before	After		Lower bound	Upper bound		
Hunger pains	2.45 ± 1.68	1.68 ± 1.09	−0.53	−1.010	−0.059	2.51	0.020*
Gastrectasia	3.64 ± 2.19	2.64 ± 1.59	−0.79	−1.296	−0.276	3.69	0.001*
Constipation	2.59 ± 1.94	1.77 ± 1.34	−0.56	−1.035	−0.079	2.61	0.016*
Total score	36.23 ± 15.02	29.82 ± 12.03	−0.64	−1.126	−0.150	2.99	0.007*

* p < 0.05. Only p < 0.05 result showed in this table.

Subsequently, we conducted a Multiple Linear Regression to assess how background characteristics influenced the effects of the Kampo formulas on the 16 gastrointestinal symptoms measured by the GSRS. Multiple linear regression revealed that the overall regression model for burping did not reach statistical significance (F (10,11) = 1.23, p = 0.374). However, smoking habits remained a significant predictor of the burping score (R^2^ = 0.576, β = −0.768, p = 0.027). The overall regression model for diarrhea did not reach statistical significance (F (10,11) = 1.14, p = 0.424). However, marital status remained a significant predictor of the diarrhea score (R^2^ = 0.555, β = −0.619, p = 0.033). The overall regression model for loose stools did not reach statistical significance (F (10,11) = 2.28, p = 0.103). However, marital status remained a significant predictor of the loose stools score (R^2^ = 0.715, β = −0.562, p = 0.018). The overall regression model for hard stools did not reach statistical significance (F (10,11) = 2.28, p = 0.102). However, sleep quality remained a significant predictor of the hard stools score (R^2^ = 0.715, β = −0.615, p = 0.032). All the Multiple linear regression results are presented in Table 5; Supplementary Table S3.

**TABLE 5:** Multiple linear regression analysis results of the Gastrointestinal Symptom Rating Scale (GSRS).

GSRS items	Factors	R^2^	β	Std. Error	95% CI		t	P value
					Lower bound	Upper bound		
Burping	Smoking	0.576	−0.768	0.438	−2.106	−0.154	−2.58	0.027*
Diarrhea	Marriage	0.555	−0.619	0.767	−3.613	−0.194	−2.48	0.033*
Loose stools	Marriage	0.715	−0.562	0.580	−2.922	−0.338	−2.81	0.018*
Hard stools	Sleep	0.715	0.615	0.559	0.144	2.637	2.49	0.032*

* p < 0.05. Only p < 0.05 result showed in this table.

**FIGURE 3:**
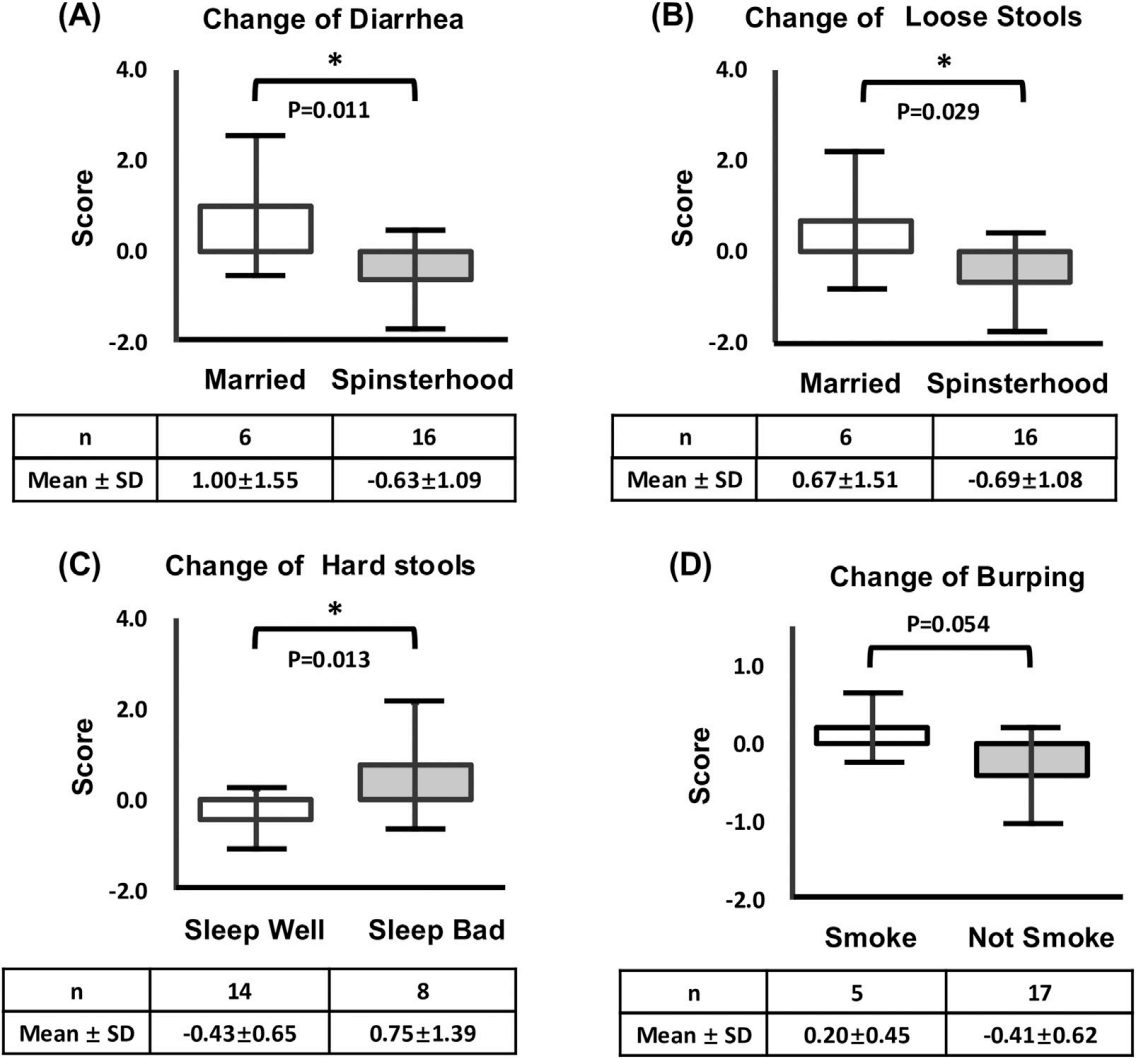
Effect of marital status on Kampo treatment–induced changes (After-Before scores) in **(A)** diarrhea, **(B)** loose stools. **(C)** Effect of sleep quality on Kampo treatment–induced changes (After-Before scores) in hard stools. **(D)** Effect of smoking habits on Kampo treatment–induced changes (After-Before scores) in Burping. *p < 0.05.

Following this, two independent-sample t-tests were conducted to evaluate changes in diarrhea, and loose stools (married vs. spinsterhood), Burping (smokers vs. nonsmokers), and hard stool (good vs. poor sleep quality). Significant differences were found in diarrhea difference by 1.63 points (95% CI [-2.423, −0.241]; Cohen’s d = −1.33, p = 0.011), and loose stools difference by 1.36 points (95% CI [-2.195, −0.063]; Cohen’s d = −1.13, p = 0.029) between married and spinsterhood. Hard stools difference by 1.18 points (95% CI [-2.219, −0.204]; Cohen’s d = −1.21, p = 0.013) between those with good and poor sleep quality, respectively. However, no significant difference was found in the Burping difference by 0.61 points (95% CI [-0.075, 2.156]; Cohen’s d = 1.04, p = 0.054) between smokers and non-smokers, and two independent-sample t-test results are provided in Table 6; Figure 3.

**TABLE 6:** Two independent sample T-tests Result of the Gastrointestinal Symptom Rating Scale (GSRS).

GSRS items	Factors	Category	F	Cohen’s d	95% CI		Mean ± SD	t	P value
					Lower bound	Upper bound			
Burping	Smoking	Yes	2.218	1.04	−0.075	2.156	0.20 ± 0.45	2.05	0.054
		No					−0.41 ± 0.62		
Diarrhea	Marriage	Married	2.945	−1.33	−2.423	−0.241	1.00 ± 1.55	2.78	0.011*
		Spinsterhood					−0.63 ± 1.09		
Loose stools	Marriage	Married	3.181	−1.13	−2.195	−0.063	0.67 ± 1.51	2.36	0.029*
		Spinsterhood					−0.69 ± 1.08		
Hard stools	Sleep	Good	1.989	−1.21	−2.219	−0.204	−0.43 ± 0.65	−2.73	0.013*
		Bad					0.75 ± 1.39		

* P < 0.05.

### 3.4 Profile of mood states second edition-adult short form (POMS 2-A)

**FIGURE 4:**
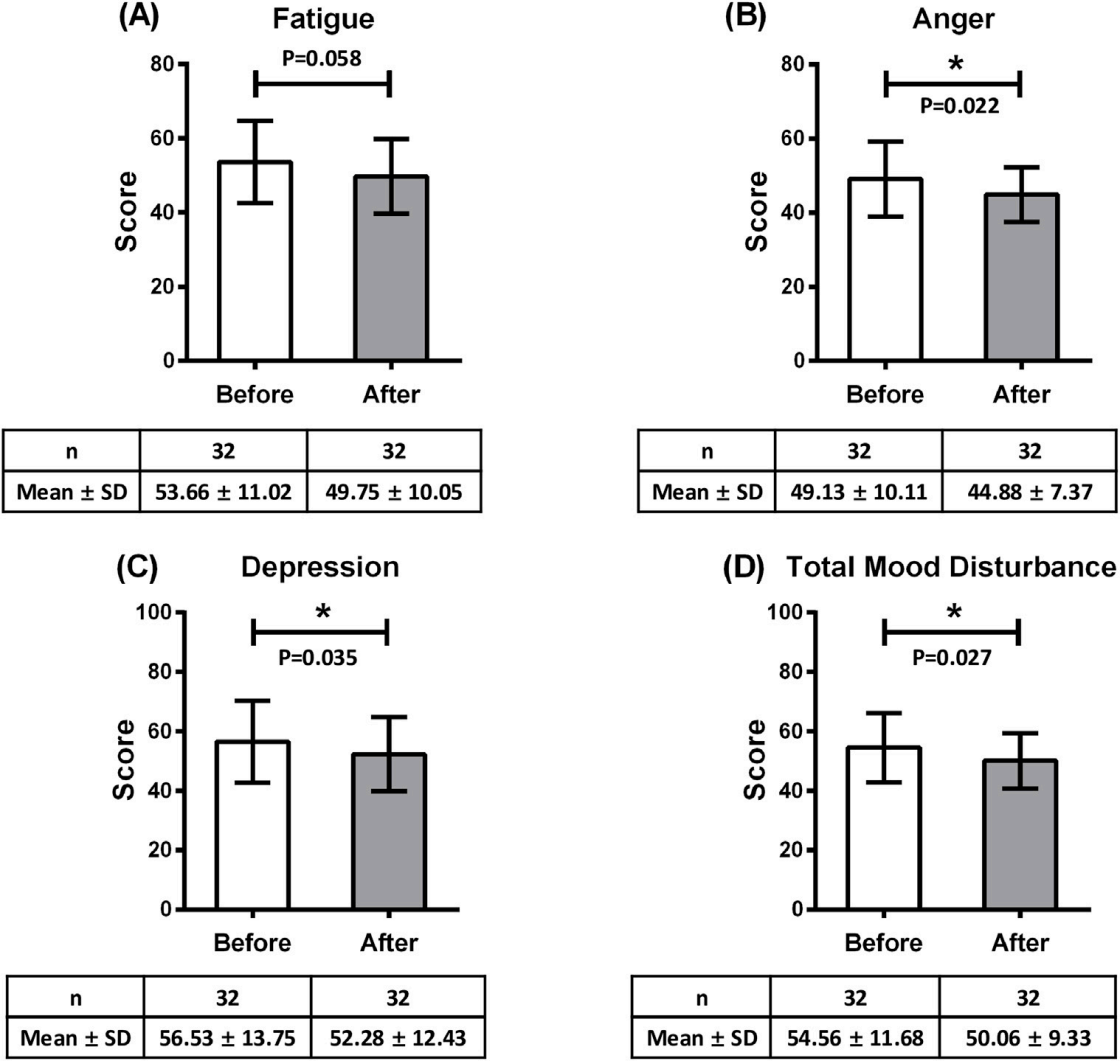
Kampo treatment–induced changes in **(A)** fatigue, **(B)** anger, **(C)** depression, **(D)** total mood disturbance. *p < 0.05.

We conducted a paired t-test to compare POMS 2-A scores before and after Kampo treatment (Table 7; Supplementary Table S7). Significant reductions were found in anger, depression and TMD scores before and after Kampo treatment. Anger decreased by 4.25 points (95% CI [-0.803, −0.049]; Cohen’s d = −0.43, p = 0.022, q = 0.070). Depression decreased by 4.25 points (95% CI [-0.764, −0.015]; Cohen’s d = −0.39, p = 0.035, q = 0.070). TMD scores decreased by 4.50 points (95% CI [-0.785, −0.033]; Cohen’s d = −0.41, p = 0.027, q = 0.070) (Figure 4). The overall responder rate for stress-relief effects under Kampo treatment was 59.4% (95% CI [43.8–75.0]). Among the specific Kampo formulas, the responder rate of bukuryoin was 58.8% (95% CI [35.3–82.4]), that of heiisan was 60.0% (95% CI [30.0–90.0]), and that of rikkunshito was 60.0% (95% CI [20.0–100.0]).

**TABLE 7:** Paired t-test Result of Profile of Mood States Second Edition-Adult Short Form (POMS 2-A).

POMS 2-A items	Mean ± SD		Cohen’s d	95% CI		t	P value
	Before	After		Lower bound	Upper bound		
Anger	49.13 ± 10.11	44.88 ± 7.37	−0.43	−0.803	−0.049	2.41	0.022*
Depression	56.53 ± 13.75	52.28 ± 12.43	−0.39	−0.764	−0.015	2.2	0.035*
Total mood disturbance	54.56 ± 11.68	50.06 ± 9.33	−0.41	−0.785	−0.033	2.31	0.027*

* p < 0.05. Only p < 0.05 result showed in this table.

Subsequently, we used Multiple linear regression to assess how background characteristics influenced the effects of the Kampo formulas on POMS 2-A scores, with mood change scores for anger, confusion, depression, fatigue, tension, vigor, friendliness, and TMD as dependent variables. However, the analysis showed that background characteristics did not significantly influence the POMS 2-A scores. All the Multiple linear regression results are presented in Supplementary Table S5.

## 4 Discussion

### 4.1 Pharmacological effect of Kampo formulas

Our findings highlight two key points: (1) GSRS scores indicated a possible improvement trend in gastrointestinal symptoms among FD patients following treatment, with lifestyle factors potentially influencing these outcomes; (2) POMS 2-A results showed improvement in stress-related symptoms, including anger, depression, and total mood disturbance. Since this was an observational study, no placebo group was included. However, we compared our findings with placebo response rates derived from a systematic review and meta-analysis on FD (Bosman et al., 2023), which incorporated 26 randomized controlled trials. This analysis showed that the pooled placebo response rate was 39.6%—a value lower than both our overall responder rate and the responder rates of each specific Kampo formula. These results suggest that Kampo treatment may have potential benefits for gastrointestinal symptoms, though differences in study design preclude firm conclusions. An expanding body of evidence underscores the intricate relationship among FD, anxiety and depression (Wang, Q.Q. et al., 2022; Carpinelli et al., 2023; Chen et al., 2023). Although several pharmacological treatments are available for FD, their efficacy, particularly for comorbid anxiety and depression, remains limited (Miwa et al., 2022). Therefore, our findings may help to inform us of more effective therapeutic strategies.

Our analysis revealed no significant differences in gastrointestinal or stress-related symptom improvement among patients treated with different Kampo formulas, possibly reflecting both the personalized selection of formulas by attending physicians based on individual symptom profiles and the substantial overlap in botanical drugs among these Kampo formulas. The common botanical drugs in the three Kampo formulas included Wolfiporia cocos Ryvarden et Gilbertson [Polyporaceae; Poria Sclerotium], Atractylodes lancea De Candolle; [Asteraceae; Atractylodes Lancea Rhizome], Citrus reticulata Blanco [Rutaceae; Citri Unshiu Pericarpium], Panax ginseng C. A. Meyer [Araliaceae; Ginseng Radix et Rhizoma], Zingiber officinale Roscoe [Zingiberaceae; Zingiberis Rhizoma], Glycyrrhiza uralensis Fischer [Fabaceae; Glycyrrhizae Radix et Rhizoma], Ziziphus jujuba Miller var. inermis Rehder [Rhamnaceae; Ziziphi Fructus].

Polysaccharides derived from Poria Sclerotium and Atractylodes Lancea Rhizome modulate the gut microbiota by enhancing intestinal barrier integrity and attenuating intestinal inflammation, potentially through the increased production of short-chain fatty acids (SCFAs) (Wang, D. et al., 2023; Lai et al., 2025). Naringenin, a major flavanone derived from Citri Unshiu Pericarpium, has been shown to regulate the NF-κB signaling pathway in Caco-2 cells (human colorectal adenocarcinoma cell line), potentially by enhancing intestinal barrier integrity and modulating gut commensal bacteria (Lee et al., 2023). Ginseng Radix et Rhizoma has been shown to effectively alleviate stress-related depression and anxiety by modulating the hypothalamic–pituitary–adrenal (HPA) axis, thereby improving the physiological response to stressful environments (Lee and Rhee, 2017). Ginseng polysaccharides have also been shown to exhibit anti-fatigue activity (Wang, J. et al., 2010). Clinical studies have demonstrated that Zingiberis Rhizoma consumption promotes relaxation of the lower esophageal sphincter and reduces esophageal contraction velocity, which may underlie its anti-flatulent effects (Haniadka et al., 2013). Gingerol has been shown to alleviate cisplatin-induced emesis in animal models, potentially via coordinated modulation of the 5-HT, SP, and dopamine pathways (Tian et al., 2020). Liquiritin is a flavonoid compound and one of the key bioactive metabolites naturally occurring in Glycyrrhizae Radix et Rhizoma. Previous studies have demonstrated that liquiritin exhibits antidepressant-like effects in a rat model of depression induced by chronic variable stress, possibly through its antioxidant properties and protective effects against oxidative stress (Zhao et al., 2008). Ziziphi Fructus fruit extract has demonstrated gastroprotective effects against ethanol-induced gastric ulcers in rats. These effects are likely attributed to the biological activity of its rich polyphenolic content (Tanyeli et al., 2023).

Based on the above, botanical drugs used in Kampo formulas may contribute to symptom relief through distinct yet complementary mechanisms. For gastrointestinal symptoms, Poria Sclerotium, Atractylodes Lancea Rhizome, Citri Unshiu Pericarpium, Zingiberis Rhizoma and Ziziphi Fructus demonstrate prokinetic, antiemetic, mucosal-protective, and anti-inflammatory effects, which correspond to the improvements in dyspeptic symptoms observed in this study. Meanwhile, Ginseng Radix et Rhizoma and Glycyrrhizae Radix et Rhizoma show antidepressant, anxiolytic, and stress-attenuating effects, likely mediated through modulation of the hypothalamic–pituitary–adrenal (HPA) axis, supporting the observed psychological improvements. Collectively, the multi-targeted pharmacological effects of botanical drugs may act synergistically to influence both gastrointestinal function and mental health in patients with FD.

### 4.2 Factors that influence kampo treatment

Our study found preliminary associations between lifestyle factors (marital status, smoking, sleep) and treatment responses; however, due to the lack of statistical significance in the regression model, these findings remain exploratory.

#### 4.2.1 Marital status

A survey conducted in southern Iran revealed that married women under the age of 35 years had a higher likelihood of experiencing FD (Masoumi et al., 2015). Marital satisfaction is a potential moderator in the relationship between stress and depression (Shi and Whisman, 2023; Han et al., 2024). In our study, we observed that single patients presented with more severe symptoms of acid reflux, diarrhea, and loose stools before Kampo treatment. However, they exhibited better therapeutic responses than married individuals. After Kampo treatment, those in spinsterhood showed lighter symptoms than married individuals. This difference may have resulted from reduced family related stress among spinsterhoods, affording them greater dietary flexibility and enhancing the efficacy of treatment.

#### 4.2.2 Sleep quality

The relationship between stress levels and sleep quality among doctors during on-call shifts is correlated with the occurrence of functional gastrointestinal disorders (Lim et al., 2017). Our results revealed that individuals with good sleep patterns displayed milder hard-stool symptoms after Kampo treatment. This difference may result from good sleep-enhancing immune function, reducing stress (Kalmbach et al., 2018) and regulating the sympathetic nervous system (Trinder et al., 2001), improving digestion, and boosting the outcomes of treatment.

#### 4.2.3 Smoking habits

In our study, nonsmokers had more severe burping symptoms before treatment but responded better to Kampo therapy. Previous studies suggested that smoking is a risk factor for a broad range of gastrointestinal diseases (Yuan et al., 2023). This difference may be explained by the fact that nonsmokers have lower exposure to smoking-related risks, whereas smokers are subject to such risks. Additionally, Multiple linear regression analysis showed a significant relationship between smoking habits and differences in Burping scores. However, subsequent independent sample t-tests showed no significant differences in the Burping scores among individuals with different smoking habits. This inconsistency may be attributed to the small sample size.

### 4.3 Limitations and future plan

Our study has several limitations. First, since this is a single-arm, exploratory observation without a concurrent placebo group, the observational design limits interpretation of the effects of Kampo treatment and their comparison with standard care. FD is characterized by a high placebo response rate. Although we compared the response rates in our study with those of placebo groups in previously published FD randomized controlled trials, differences in assessment tools and study populations cannot be ignored. As an exploratory observational study, we did not predefine effect size thresholds, so no non-inferiority/superiority analyses were performed among the formulas. This design cannot rule out a type II error, and the results should be interpreted as hypothesis-generating, particularly with respect to relative and subtype-specific efficacy. Future randomized controlled trials are warranted to establish the definitive efficacy of Kampo treatment. Second, the small sample size and incomplete background information (e.g., *H. pylori* status and FD subtype) constrain causal inference and reduce the interpretability of inter-formula comparisons. Third, Kampo prescriptions from a single manufacturer (Tsumura) may limit generalizability. In addition, this study was conducted during the COVID-19 pandemic, all patients were outpatient, resulting in follow-up challenges and a high dropout rate. Moreover, some older patients failed to return complete questionnaires, contributing to missing data. Finally, this study relied only on two subjective evaluation items. Future studies should incorporate objective evaluation measures, such as gastric motility tests, autonomic function indices, or stress-related biomarkers, to enhance the credibility of the findings.

In summary, future studies should be designed to address these limitations by conducting larger, multicenter randomized controlled trials with predefined statistical power, incorporating important covariates such as *H. pylori* infection status and psychological factors, and implementing comprehensive safety monitoring to confirm and extend the present findings.

## 5 Conclusion

This exploratory observational study suggests potential improvements in gastrointestinal symptoms and stress levels in FD patients following Kampo treatment. No statistically significant differences were observed among the formulas; however, the study was not powered to determine equivalence or non-inferiority. Additionally, marital status, smoking habits, and sleep quality may be associated with the outcomes of Kampo treatment, which could serve as avenues for further investigation.

## Data Availability

The raw data supporting the conclusions of this article will be made available by the authors, without undue reservation.

## Abbreviations

FD, Functional Dyspepsia; GSRS, Gastrointestinal Symptom Rating Scale; POMS-2A, Profile of Mood States Second Edition-Adult Short Form.
