# Supplementary material for: Integrated effects of Kampo treatment on gastrointestinal symptoms and stress in patients with functional dyspepsia: a preliminary prospective observational study
Source: Front Pharmacol. 2025 Nov 21;16:1685656. doi: 10.3389/fphar.2025.1685656 (PMC12678293; doi:10.3389/fphar.2025.1685656)
Supplement: Supplementary file 1 [file Table2.docx]

**Supplementary Table 1. Patient demographic characteristics (Supplementary).**

|  | Number(%) |
| --- | --- |
| Gender |  |
| Men | 14(42.4) |
| Women | 19(57.6) |
| Occupation |  |
| With | 14(42.4) |
| Without | 19(57.6) |
| Marriage |  |
| Married | 10(30.3) |
| Spinsterhood | 23(69.7) |
| Sleep |  |
| Good | 21(63.6) |
| Bad | 12(36.4) |
| Appetite |  |
| Good | 25(75.8) |
| Bad | 8(24.2) |
| Smoking |  |
| Yes | 8(24.2) |
| No | 25(75.8) |
| Drinking |  |
| Yes | 14(42.4) |
| No | 19(57.6) |
| Kampo |  |
| Bukuryoin | 17(51.5) |
| Rikkunshito | 6(18.2) |
| Heiisan | 10(30.3) |

**Supplementary Table 2. One way ANOVA results of the Gastrointestinal Symptom Rating Scale.**

| **GSRS items** | **Kampo** | **n** | **Mean** | **SD** | **95% CI** | | **F** | **Effect Size** | **P**  **value** |
| --- | --- | --- | --- | --- | --- | --- | --- | --- | --- |
|  |  |  |  |  | **Lower Bound** | **Upper Bound** |  |  |  |
| Stomachache | Bukuryoin | 9 | -1.11 | 2.37 | -2.932 | 0.710 | 0.635 | 0.063 | 0.541 |
|  | Rikkunshito | 5 | 0.00 | 1.22 | -1.521 | 1.521 |  |  |  |
|  | Heiisan | 8 | -0.50 | 1.31 | -1.595 | 0.595 |  |  |  |
| Heartburn | Bukuryoin | 9 | -0.11 | 1.76 | -1.467 | 1.245 | 0.791 | 0.077 | 0.468 |
|  | Rikkunshito | 5 | 0.20 | 0.84 | -0.839 | 1.239 |  |  |  |
|  | Heiisan | 8 | -0.88 | 1.81 | -2.386 | 0.636 |  |  |  |
| Acid Reflux | Bukuryoin | 9 | -0.44 | 1.24 | -1.395 | 0.506 | 1.903 | 0.167 | 0.177 |
|  | Rikkunshito | 5 | 0.60 | 0.89 | -0.511 | 1.711 |  |  |  |
|  | Heiisan | 8 | -1.00 | 1.85 | -2.548 | 0.548 |  |  |  |
| Hunger Pains | Bukuryoin | 9 | -1.11 | 1.05 | -1.921 | -0.301 | 0.526 | 0.052 | 0.600 |
|  | Rikkunshito | 5 | -0.80 | 1.10 | -2.160 | 0.560 |  |  |  |
|  | Heiisan | 8 | -0.38 | 2.00 | -2.043 | 1.293 |  |  |  |
| Nausea | Bukuryoin | 9 | -0.67 | 1.12 | -1.526 | 0.193 | 1.790 | 0.159 | 0.194 |
|  | Rikkunshito | 5 | 0.40 | 1.52 | -1.483 | 2.283 |  |  |  |
|  | Heiisan | 8 | -1.00 | 1.41 | -2.182 | 0.182 |  |  |  |
| Rumbling | Bukuryoin | 9 | 0.00 | 1.12 | -0.859 | 0.859 | 0.884 | 0.085 | 0.429 |
|  | Rikkunshito | 5 | 0.20 | 0.84 | -0.839 | 1.239 |  |  |  |
|  | Heiisan | 8 | -0.75 | 1.91 | -2.346 | 0.846 |  |  |  |
| Gastrectasia | Bukuryoin | 9 | -1.11 | 1.54 | -2.292 | 0.070 | 0.088 | 0.009 | 0.916 |
|  | Rikkunshito | 5 | -0.80 | 1.30 | -2.419 | 0.819 |  |  |  |
|  | Heiisan | 8 | -1.00 | 1.07 | -1.894 | -0.106 |  |  |  |
| Burp | Bukuryoin | 9 | -0.33 | 0.87 | -0.999 | 0.332 | 0.073 | 0.008 | 0.930 |
|  | Rikkunshito | 5 | -0.20 | 0.45 | -0.755 | 0.355 |  |  |  |
|  | Heiisan | 8 | -0.25 | 0.46 | -0.637 | 0.137 |  |  |  |
| Fart | Bukuryoin | 9 | -0.33 | 1.66 | -1.608 | 0.941 | 0.085 | 0.009 | 0.918 |
|  | Rikkunshito | 5 | -0.40 | 0.89 | -1.511 | 0.711 |  |  |  |
|  | Heiisan | 8 | -0.13 | 0.99 | -0.954 | 0.704 |  |  |  |
| Constipation | Bukuryoin | 9 | -0.33 | 1.22 | -1.275 | 0.608 | 1.074 | 0.102 | 0.362 |
|  | Rikkunshito | 5 | -0.80 | 1.79 | -3.021 | 1.421 |  |  |  |
|  | Heiisan | 8 | -1.38 | 1.51 | -2.634 | -0.116 |  |  |  |
| Diarrhea | Bukuryoin | 9 | 0.00 | 1.22 | -0.941 | 0.941 | 0.284 | 0.029 | 0.756 |
|  | Rikkunshito | 5 | -0.60 | 0.55 | -1.280 | 0.080 |  |  |  |
|  | Heiisan | 8 | -0.13 | 1.96 | -1.763 | 1.513 |  |  |  |
| Loose stools | Bukuryoin | 9 | -0.67 | 1.58 | -1.882 | 0.549 | 1.183 | 0.111 | 0.328 |
|  | Rikkunshito | 5 | -0.60 | 0.55 | -1.280 | 0.080 |  |  |  |
|  | Heiisan | 8 | 0.25 | 1.28 | -0.822 | 1.322 |  |  |  |
| Hard stools | Bukuryoin | 9 | 0.33 | 1.58 | -0.882 | 1.549 | 0.760 | 0.074 | 0.482 |
|  | Rikkunshito | 5 | -0.40 | 0.55 | -1.080 | 0.280 |  |  |  |
|  | Heiisan | 8 | -0.13 | 0.64 | -0.661 | 0.411 |  |  |  |
| Urgent need for defecation | Bukuryoin | 9 | -0.56 | 2.55 | -2.520 | 1.408 | 0.329 | 0.033 | 0.724 |
|  | Rikkunshito | 5 | 0.20 | 0.84 | -0.839 | 1.239 |  |  |  |
|  | Heiisan | 8 | 0.00 | 1.20 | -0.999 | 0.999 |  |  |  |
| Incomplete evacuation feeling | Bukuryoin | 9 | -0.56 | 1.24 | -1.506 | 0.395 | 0.338 | 0.034 | 0.717 |
|  | Rikkunshito | 5 | 0.00 | 0.71 | -0.878 | 0.878 |  |  |  |
|  | Heiisan | 8 | -0.63 | 1.85 | -2.169 | 0.919 |  |  |  |
| Total score | Bukuryoin | 9 | -7.00 | 9.29 | -14.139 | 0.139 | 0.365 | 0.037 | 0.699 |
|  | Rikkunshito | 5 | -3.00 | 8.94 | -14.106 | 8.106 |  |  |  |
|  | Heiisan | 8 | -7.88 | 12.14 | -18.020 | 2.270 |  |  |  |

*P<0.05

GSRS:Gastrointestinal Symptom Rating Scale.

**Supplementary Table 3. Multiple linear regression analysis results of the Gastrointestinal Symptom Rating Scale (Supplementary).**

| **GSRS items** | **Factors** | **R²** | **β** | **Std. Error** | **95% CI** | | **t** | **P**  **value** |
| --- | --- | --- | --- | --- | --- | --- | --- | --- |
|  |  |  |  |  | **Lower Bound** | **Upper Bound** |  |  |
| Stomachache | Gender | 0.220 | 0.334 | 1.777 | -2.748 | 5.173 | 0.68 | 0.511 |
|  | Age |  | 0.179 | 0.051 | -0.097 | 0.131 | 0.34 | 0.743 |
|  | BMI |  | 0.050 | 0.217 | -0.459 | 0.506 | 0.11 | 0.915 |
|  | Occupation |  | 0.177 | 1.432 | -2.561 | 3.821 | 0.44 | 0.669 |
|  | Marriage |  | 0.109 | 1.296 | -2.462 | 3.313 | 0.33 | 0.749 |
|  | Sleep |  | -0.113 | 1.487 | -3.723 | 2.903 | -0.28 | 0.788 |
|  | Appetite |  | 0.131 | 1.580 | -3.006 | 4.035 | 0.33 | 0.752 |
|  | Smoke |  | 0.056 | 1.682 | -3.517 | 3.980 | 0.14 | 0.893 |
|  | Drink |  | -0.116 | 2.120 | -5.144 | 4.303 | -0.20 | 0.847 |
|  | Bukuryoin |  | -0.321 | 1.478 | -4.431 | 2.154 | -0.77 | 0.459 |
|  | Heiisan |  | -0.125 | 1.653 | -4.135 | 3.231 | -0.27 | 0.790 |
| Heartburn | Gender | 0.418 | -0.035 | 1.387 | -3.207 | 2.975 | -0.08 | 0.935 |
|  | Age |  | -0.026 | 0.040 | -0.091 | 0.087 | -0.06 | 0.956 |
|  | BMI |  | -0.024 | 0.169 | -0.387 | 0.366 | -0.06 | 0.952 |
|  | Occupation |  | 0.080 | 1.118 | -2.233 | 2.747 | 0.23 | 0.823 |
|  | Marriage |  | -0.298 | 1.011 | -3.309 | 1.198 | -1.04 | 0.321 |
|  | Sleep |  | 0.256 | 1.160 | -1.746 | 3.425 | 0.72 | 0.486 |
|  | Appetite |  | 0.036 | 1.233 | -2.620 | 2.875 | 0.10 | 0.920 |
|  | Smoke |  | 0.458 | 1.313 | -1.200 | 4.651 | 1.31 | 0.218 |
|  | Drink |  | -0.137 | 1.655 | -4.135 | 3.239 | -0.27 | 0.792 |
|  | Bukuryoin |  | -0.237 | 1.153 | -3.329 | 1.810 | -0.66 | 0.525 |
|  | Heiisan |  | -0.392 | 1.290 | -4.159 | 1.590 | -1.00 | 0.343 |
| Acid Reflux | Gender | 0.454 | 0.064 | 1.249 | -2.588 | 2.976 | 0.16 | 0.879 |
|  | Age |  | 0.113 | 0.036 | -0.071 | 0.089 | 0.25 | 0.805 |
|  | BMI |  | -0.001 | 0.152 | -0.340 | 0.339 | 0.00 | 0.997 |
|  | Occupation |  | 0.159 | 1.006 | -1.769 | 2.714 | 0.47 | 0.648 |
|  | Marriage |  | -0.449 | 0.910 | -3.507 | 0.550 | -1.62 | 0.135 |
|  | Sleep |  | 0.131 | 1.045 | -1.929 | 2.726 | 0.38 | 0.711 |
|  | Appetite |  | -0.161 | 1.110 | -3.003 | 1.943 | -0.48 | 0.643 |
|  | Smoke |  | 0.086 | 1.182 | -2.334 | 2.933 | 0.25 | 0.805 |
|  | Drink |  | -0.040 | 1.489 | -3.440 | 3.197 | -0.08 | 0.937 |
|  | Bukuryoin |  | -0.467 | 1.038 | -3.706 | 0.920 | -1.34 | 0.209 |
|  | Heiisan |  | -0.647 | 1.161 | -4.561 | 0.614 | -1.70 | 0.120 |
| Hunger Pains | Gender | 0.179 | -0.194 | 1.474 | -3.854 | 2.716 | -0.39 | 0.708 |
|  | Age |  | -0.141 | 0.042 | -0.106 | 0.084 | -0.26 | 0.801 |
|  | BMI |  | -0.177 | 0.180 | -0.468 | 0.333 | -0.38 | 0.716 |
|  | Occupation |  | -0.093 | 1.188 | -2.913 | 2.381 | -0.22 | 0.827 |
|  | Marriage |  | -0.256 | 1.075 | -3.206 | 1.584 | -0.75 | 0.468 |
|  | Sleep |  | 0.170 | 1.233 | -2.249 | 3.248 | 0.41 | 0.694 |
|  | Appetite |  | 0.166 | 1.311 | -2.394 | 3.448 | 0.40 | 0.696 |
|  | Smoke |  | 0.086 | 1.396 | -2.819 | 3.400 | 0.21 | 0.839 |
|  | Drink |  | -0.055 | 1.759 | -4.080 | 3.757 | -0.09 | 0.929 |
|  | Bukuryoin |  | -0.138 | 1.226 | -3.127 | 2.336 | -0.32 | 0.754 |
|  | Heiisan |  | 0.243 | 1.371 | -2.343 | 3.768 | 0.52 | 0.615 |
| Nausea | Gender | 0.549 | -0.452 | 1.037 | -3.570 | 1.051 | -1.22 | 0.252 |
|  | Age |  | -0.481 | 0.030 | -0.102 | 0.031 | -1.19 | 0.262 |
|  | BMI |  | -0.505 | 0.126 | -0.464 | 0.099 | -1.45 | 0.179 |
|  | Occupation |  | -0.359 | 0.835 | -2.839 | 0.884 | -1.17 | 0.269 |
|  | Marriage |  | -0.103 | 0.756 | -1.994 | 1.375 | -0.41 | 0.691 |
|  | Sleep |  | 0.345 | 0.867 | -0.972 | 2.893 | 1.11 | 0.294 |
|  | Appetite |  | 0.305 | 0.922 | -1.137 | 2.970 | 1.00 | 0.343 |
|  | Smoke |  | 0.135 | 0.981 | -1.757 | 2.616 | 0.44 | 0.671 |
|  | Drink |  | -0.313 | 1.237 | -3.627 | 1.884 | -0.71 | 0.497 |
|  | Bukuryoin |  | -0.434 | 0.862 | -3.103 | 0.739 | -1.37 | 0.200 |
|  | Heiisan |  | -0.217 | 0.964 | -2.754 | 1.543 | -0.63 | 0.544 |
| Rumbling | Gender | 0.412 | -0.126 | 1.219 | -3.078 | 2.354 | -0.30 | 0.773 |
|  | Age |  | 0.046 | 0.035 | -0.075 | 0.082 | 0.10 | 0.923 |
|  | BMI |  | -0.356 | 0.149 | -0.464 | 0.198 | -0.89 | 0.393 |
|  | Occupation |  | 0.085 | 0.982 | -1.951 | 2.426 | 0.24 | 0.814 |
|  | Marriage |  | 0.346 | 0.889 | -0.909 | 3.052 | 1.21 | 0.256 |
|  | Sleep |  | 0.063 | 1.020 | -2.093 | 2.451 | 0.18 | 0.864 |
|  | Appetite |  | 0.279 | 1.084 | -1.550 | 3.279 | 0.80 | 0.444 |
|  | Smoke |  | 0.029 | 1.154 | -2.475 | 2.667 | 0.08 | 0.935 |
|  | Drink |  | -0.403 | 1.454 | -4.396 | 2.084 | -0.80 | 0.445 |
|  | Bukuryoin |  | -0.028 | 1.014 | -2.338 | 2.179 | -0.08 | 0.939 |
|  | Heiisan |  | -0.082 | 1.134 | -2.761 | 2.291 | -0.21 | 0.840 |
| Gastrectasia | Gender | 0.456 | -0.064 | 1.057 | -2.521 | 2.189 | -0.16 | 0.878 |
|  | Age |  | -0.089 | 0.030 | -0.074 | 0.062 | -0.20 | 0.845 |
|  | BMI |  | -0.674 | 0.129 | -0.513 | 0.061 | -1.76 | 0.110 |
|  | Occupation |  | 0.138 | 0.852 | -1.548 | 2.247 | 0.41 | 0.690 |
|  | Marriage |  | 0.130 | 0.771 | -1.353 | 2.081 | 0.47 | 0.647 |
|  | Sleep |  | 0.053 | 0.884 | -1.832 | 2.108 | 0.16 | 0.879 |
|  | Appetite |  | -0.425 | 0.940 | -3.279 | 0.908 | -1.26 | 0.236 |
|  | Smoke |  | -0.413 | 1.000 | -3.455 | 1.003 | -1.23 | 0.248 |
|  | Drink |  | 0.111 | 1.261 | -2.523 | 3.095 | 0.23 | 0.825 |
|  | Bukuryoin |  | 0.095 | 0.879 | -1.717 | 2.199 | 0.27 | 0.789 |
|  | Heiisan |  | 0.049 | 0.983 | -2.064 | 2.317 | 0.13 | 0.900 |
| Burp | Gender | 0.576 | -0.034 | 0.463 | -1.075 | 0.987 | -0.10 | 0.927 |
|  | Age |  | 0.100 | 0.013 | -0.026 | 0.033 | 0.26 | 0.804 |
|  | BMI |  | -0.486 | 0.056 | -0.206 | 0.045 | -1.43 | 0.183 |
|  | Occupation |  | -0.077 | 0.373 | -0.928 | 0.734 | -0.26 | 0.800 |
|  | Marriage |  | -0.270 | 0.337 | -1.126 | 0.377 | -1.11 | 0.293 |
|  | Sleep |  | 0.319 | 0.387 | -0.453 | 1.272 | 1.06 | 0.315 |
|  | Appetite |  | -0.221 | 0.411 | -1.223 | 0.610 | -0.75 | 0.474 |
|  | Drink |  | 0.257 | 0.552 | -0.900 | 1.560 | 0.60 | 0.563 |
|  | Bukuryoin |  | 0.017 | 0.385 | -0.836 | 0.879 | 0.06 | 0.956 |
|  | Heiisan |  | 0.136 | 0.430 | -0.785 | 1.133 | 0.40 | 0.695 |
| Fart | Gender | 0.387 | -0.521 | 1.094 | -3.753 | 1.123 | -1.20 | 0.257 |
|  | Age |  | -0.430 | 0.032 | -0.099 | 0.042 | -0.91 | 0.384 |
|  | BMI |  | 0.249 | 0.133 | -0.216 | 0.378 | 0.61 | 0.556 |
|  | Occupation |  | -0.267 | 0.882 | -2.623 | 1.305 | -0.75 | 0.472 |
|  | Marriage |  | 0.033 | 0.798 | -1.687 | 1.868 | 0.11 | 0.912 |
|  | Sleep |  | 0.256 | 0.915 | -1.393 | 2.686 | 0.71 | 0.496 |
|  | Appetite |  | 0.636 | 0.973 | -0.437 | 3.898 | 1.78 | 0.106 |
|  | Smoke |  | 0.075 | 1.036 | -2.090 | 2.526 | 0.21 | 0.838 |
|  | Drink |  | 0.012 | 1.305 | -2.877 | 2.939 | 0.02 | 0.981 |
|  | Bukuryoin |  | -0.080 | 0.910 | -2.225 | 1.829 | -0.22 | 0.832 |
|  | Heiisan |  | 0.094 | 1.018 | -2.031 | 2.504 | 0.23 | 0.821 |
| Constipation | Gender | 0.407 | 0.361 | 1.273 | -1.758 | 3.913 | 0.85 | 0.417 |
|  | Age |  | 0.321 | 0.037 | -0.056 | 0.107 | 0.69 | 0.505 |
|  | BMI |  | -0.265 | 0.155 | -0.448 | 0.243 | -0.66 | 0.523 |
|  | Occupation |  | -0.049 | 1.025 | -2.428 | 2.142 | -0.14 | 0.892 |
|  | Marriage |  | -0.225 | 0.928 | -2.792 | 1.343 | -0.78 | 0.453 |
|  | Sleep |  | 0.298 | 1.065 | -1.484 | 3.261 | 0.84 | 0.423 |
|  | Appetite |  | -0.333 | 1.131 | -3.595 | 1.447 | -0.95 | 0.365 |
|  | Smoke |  | -0.371 | 1.205 | -3.953 | 1.415 | -1.05 | 0.317 |
|  | Drink |  | 0.102 | 1.518 | -3.080 | 3.686 | 0.20 | 0.846 |
|  | Bukuryoin |  | 0.171 | 1.058 | -1.859 | 2.856 | 0.47 | 0.648 |
|  | Heiisan |  | -0.088 | 1.184 | -2.901 | 2.374 | -0.22 | 0.828 |
| Diarrhea | Gender | 0.555 | -0.210 | 1.052 | -2.942 | 1.748 | -0.57 | 0.583 |
|  | Age |  | 0.100 | 0.030 | -0.060 | 0.075 | 0.25 | 0.809 |
|  | BMI |  | 0.334 | 0.128 | -0.162 | 0.409 | 0.96 | 0.358 |
|  | Occupation |  | -0.124 | 0.848 | -2.235 | 1.543 | -0.41 | 0.692 |
|  | Sleep |  | 0.368 | 0.880 | -0.915 | 3.009 | 1.19 | 0.262 |
|  | Appetite |  | -0.022 | 0.936 | -2.154 | 2.016 | -0.07 | 0.943 |
|  | Smoke |  | -0.248 | 0.996 | -3.029 | 1.410 | -0.81 | 0.435 |
|  | Drink |  | 0.461 | 1.255 | -1.484 | 4.110 | 1.05 | 0.320 |
|  | Bukuryoin |  | 0.113 | 0.875 | -1.636 | 2.264 | 0.36 | 0.727 |
|  | Heiisan |  | -0.011 | 0.979 | -2.212 | 2.150 | -0.03 | 0.976 |
| Loose stools | Gender | 0.715 | -0.251 | 0.795 | -2.446 | 1.099 | -0.85 | 0.417 |
|  | Age |  | -0.019 | 0.023 | -0.052 | 0.050 | -0.06 | 0.953 |
|  | BMI |  | -0.192 | 0.097 | -0.283 | 0.149 | -0.69 | 0.505 |
|  | Occupation |  | -0.400 | 0.641 | -2.479 | 0.377 | -1.64 | 0.132 |
|  | Sleep |  | 0.457 | 0.665 | -0.253 | 2.712 | 1.85 | 0.094 |
|  | Appetite |  | -0.276 | 0.707 | -2.376 | 0.776 | -1.13 | 0.284 |
|  | Smoke |  | -0.408 | 0.753 | -2.938 | 0.418 | -1.67 | 0.125 |
|  | Drink |  | 0.071 | 0.949 | -1.923 | 2.306 | 0.20 | 0.844 |
|  | Bukuryoin |  | -0.130 | 0.661 | -1.816 | 1.132 | -0.52 | 0.616 |
|  | Heiisan |  | 0.394 | 0.740 | -0.589 | 2.708 | 1.43 | 0.183 |
| Hard stools | Gender | 0.715 | -0.484 | 0.669 | -2.584 | 0.395 | -1.64 | 0.133 |
|  | Age |  | -0.481 | 0.019 | -0.072 | 0.014 | -1.49 | 0.166 |
|  | BMI |  | -0.153 | 0.082 | -0.226 | 0.137 | -0.55 | 0.595 |
|  | Occupation |  | -0.354 | 0.539 | -1.983 | 0.418 | -1.45 | 0.177 |
|  | Marriage |  | -0.388 | 0.488 | -2.032 | 0.140 | -1.94 | 0.081 |
|  | Appetite |  | 0.087 | 0.594 | -1.112 | 1.537 | 0.36 | 0.728 |
|  | Smoke |  | -0.066 | 0.633 | -1.581 | 1.239 | -0.27 | 0.793 |
|  | Drink |  | 0.065 | 0.798 | -1.629 | 1.925 | 0.19 | 0.857 |
|  | Bukuryoin |  | 0.178 | 0.556 | -0.845 | 1.633 | 0.71 | 0.495 |
|  | Heiisan |  | 0.092 | 0.622 | -1.177 | 1.594 | 0.34 | 0.744 |
| Urgent need for defecation | Gender | 0.574 | -0.633 | 1.315 | -5.232 | 0.630 | -1.75 | 0.111 |
|  | Age |  | -0.362 | 0.038 | -0.119 | 0.050 | -0.92 | 0.379 |
|  | BMI |  | 0.010 | 0.160 | -0.353 | 0.362 | 0.03 | 0.977 |
|  | Occupation |  | -0.165 | 1.060 | -2.947 | 1.776 | -0.55 | 0.593 |
|  | Marriage |  | -0.365 | 0.959 | -3.568 | 0.706 | -1.49 | 0.166 |
|  | Sleep |  | 0.539 | 1.101 | -0.493 | 4.412 | 1.78 | 0.105 |
|  | Appetite |  | -0.031 | 1.169 | -2.727 | 2.484 | -0.10 | 0.919 |
|  | Smoke |  | -0.289 | 1.245 | -3.978 | 1.570 | -0.97 | 0.356 |
|  | Drink |  | 0.269 | 1.569 | -2.520 | 4.473 | 0.62 | 0.548 |
|  | Bukuryoin |  | -0.320 | 1.094 | -3.575 | 1.299 | -1.04 | 0.323 |
|  | Heiisan |  | -0.235 | 1.224 | -3.581 | 1.872 | -0.70 | 0.501 |
| Incomplete evacuation feeling | Gender | 0.381 | 0.066 | 1.214 | -2.520 | 2.889 | 0.15 | 0.882 |
|  | Age |  | 0.077 | 0.035 | -0.072 | 0.084 | 0.16 | 0.875 |
|  | BMI |  | -0.433 | 0.148 | -0.486 | 0.173 | -1.06 | 0.315 |
|  | Occupation |  | 0.261 | 0.978 | -1.468 | 2.890 | 0.73 | 0.484 |
|  | Marriage |  | -0.103 | 0.885 | -2.282 | 1.662 | -0.35 | 0.733 |
|  | Sleep |  | -0.022 | 1.015 | -2.325 | 2.200 | -0.06 | 0.952 |
|  | Appetite |  | -0.270 | 1.079 | -3.217 | 1.592 | -0.75 | 0.469 |
|  | Smoke |  | -0.072 | 1.149 | -2.791 | 2.328 | -0.20 | 0.844 |
|  | Drink |  | 0.337 | 1.448 | -2.287 | 4.165 | 0.65 | 0.531 |
|  | Bukuryoin |  | 0.030 | 1.009 | -2.168 | 2.330 | 0.08 | 0.938 |
|  | Heiisan |  | -0.149 | 1.129 | -2.931 | 2.100 | -0.37 | 0.720 |
| Total score | Gender | 0.434 | -0.286 | 8.507 | -24.783 | 13.127 | -0.69 | 0.509 |
|  | Age |  | -0.142 | 0.245 | -0.623 | 0.469 | -0.31 | 0.760 |
|  | BMI |  | -0.316 | 1.037 | -3.148 | 1.472 | -0.81 | 0.438 |
|  | Occupation |  | -0.113 | 6.854 | -17.522 | 13.023 | -0.33 | 0.750 |
|  | Marriage |  | -0.409 | 6.203 | -22.845 | 4.796 | -1.46 | 0.176 |
|  | Sleep |  | 0.496 | 7.117 | -5.742 | 25.972 | 1.42 | 0.186 |
|  | Appetite |  | 0.000 | 7.563 | -16.856 | 16.846 | 0.00 | 0.999 |
|  | Smoke |  | -0.171 | 8.052 | -21.950 | 13.932 | -0.50 | 0.629 |
|  | Drink |  | 0.066 | 10.148 | -21.271 | 23.950 | 0.13 | 0.898 |
|  | Bukuryoin |  | -0.254 | 7.073 | -20.835 | 10.685 | -0.72 | 0.490 |
|  | Heiisan |  | -0.176 | 7.912 | -21.227 | 14.032 | -0.46 | 0.659 |

*P<0.05

Only p > 0.05 result showed in this table.

GSRS:Gastrointestinal Symptom Rating Scale.

**Supplementary Table 4. One way ANOVA Result of Profile of Mood States Second Edition-Adult Short Form.**

| **POMS-2A items** | **Kampo** | **n** | **Mean** | **SD** | **95% CI** | | **F** | **Effect Size** | **P**  **value** |
| --- | --- | --- | --- | --- | --- | --- | --- | --- | --- |
|  |  |  |  |  | **Lower Bound** | **Upper Bound** |  |  |  |
| Friendliness | Bukuryoin | 17 | -2.47 | 10.16 | -7.692 | 2.751 | 0.053 | 0.004 | 0.949 |
|  | Rikkunshito | 5 | -4.00 | 9.87 | -16.260 | 8.260 |  |  |  |
|  | Heiisan | 10 | -3.30 | 9.96 | -10.422 | 3.822 |  |  |  |
| Tension | Bukuryoin | 17 | -3.82 | 13.94 | -10.990 | 3.343 | 0.126 | 0.009 | 0.882 |
|  | Rikkunshito | 5 | -0.60 | 4.77 | -6.529 | 5.329 |  |  |  |
|  | Heiisan | 10 | -3.70 | 13.78 | -13.561 | 6.161 |  |  |  |
| Anger | Bukuryoin | 17 | -3.12 | 8.18 | -7.322 | 1.087 | 0.222 | 0.015 | 0.802 |
|  | Rikkunshito | 5 | -5.40 | 16.21 | -25.529 | 14.729 |  |  |  |
|  | Heiisan | 10 | -5.60 | 10.11 | -12.834 | 1.634 |  |  |  |
| Fatigue | Bukuryoin | 17 | -3.35 | 11.43 | -9.229 | 2.523 | 0.195 | 0.013 | 0.824 |
|  | Rikkunshito | 5 | -2.20 | 9.34 | -13.795 | 9.395 |  |  |  |
|  | Heiisan | 10 | -5.70 | 12.58 | -14.699 | 3.299 |  |  |  |
| Vigor | Bukuryoin | 17 | -0.59 | 6.72 | -4.042 | 2.866 | 0.011 | 0.001 | 0.990 |
|  | Rikkunshito | 5 | 0.00 | 8.75 | -10.860 | 10.860 |  |  |  |
|  | Heiisan | 10 | -0.30 | 10.75 | -7.990 | 7.390 |  |  |  |
| Confusion | Bukuryoin | 17 | -2.82 | 13.17 | -9.597 | 3.949 | 0.474 | 0.032 | 0.627 |
|  | Rikkunshito | 5 | 0.80 | 8.04 | -9.188 | 10.788 |  |  |  |
|  | Heiisan | 10 | -6.20 | 15.71 | -17.439 | 5.039 |  |  |  |
| Depression | Bukuryoin | 17 | -3.59 | 9.76 | -8.606 | 1.430 | 0.067 | 0.005 | 0.935 |
|  | Rikkunshito | 5 | -4.60 | 12.54 | -20.173 | 10.973 |  |  |  |
|  | Heiisan | 10 | -5.20 | 12.99 | -14.495 | 4.095 |  |  |  |
| Total mood disturbance | Bukuryoin | 17 | -3.82 | 10.53 | -9.235 | 1.588 | 0.191 | 0.013 | 0.828 |
|  | Rikkunshito | 5 | -3.20 | 7.33 | -12.299 | 5.899 |  |  |  |
|  | Heiisan | 10 | -6.30 | 13.80 | -16.172 | 3.572 |  |  |  |

*P<0.05

**Supplementary Table 5. Multiple linear regression analysis results of Profile of Mood States Second Edition-Adult Short Form (Supplementary).**

| **POMS-2A items** | **Factors** | **R²** | **β** | **Std. Error** | **95% CI** | | **t** | **p value** |
| --- | --- | --- | --- | --- | --- | --- | --- | --- |
|  |  |  |  |  | **Lower Bound** | **Upper Bound** |  |  |
| Friendliness | BMI | 0.185 | -0.298 | 0.646 | -2.152 | 0.527 | -1.26 | 0.222 |
|  | Occupation |  | 0.060 | 4.203 | -7.559 | 9.873 | 0.28 | 0.786 |
|  | Marriage |  | 0.123 | 4.416 | -6.532 | 11.783 | 0.60 | 0.558 |
|  | Sleep |  | -0.023 | 4.655 | -10.117 | 9.189 | -0.10 | 0.921 |
|  | Appetite |  | -0.015 | 4.946 | -10.581 | 9.934 | -0.07 | 0.948 |
|  | Smoke |  | -0.372 | 5.851 | -20.370 | 3.898 | -1.41 | 0.173 |
|  | Drink |  | 0.065 | 5.251 | -9.634 | 12.147 | 0.24 | 0.813 |
|  | Bukuryoin |  | 0.330 | 6.173 | -6.458 | 19.147 | 1.03 | 0.315 |
|  | Heiisan |  | 0.269 | 7.520 | -10.026 | 21.166 | 0.74 | 0.467 |
| Tension | BMI | 0.183 | -0.154 | 0.839 | -2.285 | 1.194 | -0.65 | 0.522 |
|  | Occupation |  | -0.100 | 5.458 | -13.839 | 8.799 | -0.46 | 0.649 |
|  | Marriage |  | -0.346 | 5.734 | -21.469 | 2.316 | -1.67 | 0.109 |
|  | Sleep |  | 0.119 | 6.045 | -9.471 | 15.601 | 0.51 | 0.617 |
|  | Appetite |  | -0.168 | 6.423 | -18.154 | 8.489 | -0.75 | 0.460 |
|  | Smoke |  | 0.012 | 7.598 | -15.427 | 16.089 | 0.04 | 0.966 |
|  | Drink |  | -0.013 | 6.820 | -14.463 | 13.824 | -0.05 | 0.963 |
|  | Bukuryoin |  | -0.231 | 8.017 | -22.396 | 10.858 | -0.72 | 0.479 |
|  | Heiisan |  | -0.128 | 9.766 | -23.699 | 16.809 | -0.35 | 0.728 |
| Anger | BMI | 0.118 | -0.247 | 0.688 | -2.117 | 0.738 | -1.00 | 0.327 |
|  | Occupation |  | -0.049 | 4.480 | -10.260 | 8.320 | -0.22 | 0.831 |
|  | Marriage |  | -0.011 | 4.707 | -9.995 | 9.527 | -0.05 | 0.961 |
|  | Sleep |  | 0.208 | 4.961 | -6.059 | 14.520 | 0.85 | 0.403 |
|  | Appetite |  | -0.004 | 5.272 | -11.021 | 10.846 | -0.02 | 0.987 |
|  | Smoke |  | -0.094 | 6.236 | -15.056 | 10.810 | -0.34 | 0.737 |
|  | Drink |  | 0.044 | 5.597 | -10.727 | 12.489 | 0.16 | 0.876 |
|  | Bukuryoin |  | 0.193 | 6.580 | -9.847 | 17.446 | 0.58 | 0.570 |
|  | Heiisan |  | 0.165 | 8.016 | -13.132 | 20.115 | 0.44 | 0.667 |
| Fatigue | BMI | 0.050 | -0.034 | 0.804 | -1.775 | 1.560 | -0.13 | 0.895 |
|  | Occupation |  | 0.117 | 5.233 | -8.237 | 13.467 | 0.50 | 0.622 |
|  | Marriage |  | -0.064 | 5.498 | -12.983 | 9.821 | -0.29 | 0.776 |
|  | Sleep |  | 0.044 | 5.796 | -11.007 | 13.031 | 0.18 | 0.863 |
|  | Appetite |  | 0.029 | 6.158 | -12.044 | 13.500 | 0.12 | 0.907 |
|  | Smoke |  | 0.153 | 7.285 | -11.192 | 19.024 | 0.54 | 0.596 |
|  | Drink |  | -0.159 | 6.538 | -17.101 | 10.018 | -0.54 | 0.594 |
|  | Bukuryoin |  | -0.172 | 7.687 | -19.756 | 12.126 | -0.50 | 0.625 |
|  | Heiisan |  | -0.206 | 9.363 | -24.335 | 14.502 | -0.53 | 0.605 |
| Vigor | BMI | 0.075 | -0.104 | 0.577 | -1.435 | 0.958 | -0.41 | 0.683 |
|  | Occupation |  | 0.138 | 3.755 | -5.555 | 10.021 | 0.60 | 0.558 |
|  | Marriage |  | 0.132 | 3.945 | -5.814 | 10.551 | 0.60 | 0.554 |
|  | Sleep |  | 0.085 | 4.159 | -7.209 | 10.042 | 0.34 | 0.737 |
|  | Appetite |  | 0.132 | 4.419 | -6.718 | 11.613 | 0.55 | 0.585 |
|  | Smoke |  | 0.012 | 5.228 | -10.617 | 11.068 | 0.04 | 0.966 |
|  | Drink |  | -0.016 | 4.692 | -9.993 | 9.469 | -0.06 | 0.956 |
|  | Bukuryoin |  | -0.004 | 5.516 | -11.498 | 11.382 | -0.01 | 0.992 |
|  | Heiisan |  | 0.051 | 6.719 | -13.056 | 14.814 | 0.13 | 0.897 |
| Confusion | BMI | 0.192 | -0.205 | 0.874 | -2.572 | 1.051 | -0.87 | 0.394 |
|  | Occupation |  | -0.057 | 5.684 | -13.287 | 10.291 | -0.26 | 0.795 |
|  | Marriage |  | -0.280 | 5.972 | -20.485 | 4.287 | -1.36 | 0.189 |
|  | Sleep |  | 0.174 | 6.296 | -8.371 | 17.742 | 0.74 | 0.465 |
|  | Appetite |  | -0.146 | 6.690 | -18.255 | 9.493 | -0.66 | 0.519 |
|  | Smoke |  | 0.059 | 7.914 | -14.642 | 18.182 | 0.22 | 0.825 |
|  | Drink |  | 0.016 | 7.103 | -14.323 | 15.137 | 0.06 | 0.955 |
|  | Bukuryoin |  | -0.231 | 8.350 | -23.344 | 11.289 | -0.72 | 0.478 |
|  | Heiisan |  | -0.237 | 10.171 | -27.761 | 14.428 | -0.66 | 0.519 |
| Depression | BMI | 0.337 | -0.247 | 0.652 | -2.107 | 0.599 | -1.16 | 0.260 |
|  | Occupation |  | 0.263 | 4.244 | -3.112 | 14.490 | 1.34 | 0.194 |
|  | Marriage |  | -0.316 | 4.459 | -16.786 | 1.708 | -1.69 | 0.105 |
|  | Sleep |  | 0.186 | 4.700 | -5.628 | 13.868 | 0.88 | 0.390 |
|  | Appetite |  | -0.397 | 4.995 | -20.211 | 0.505 | -1.97 | 0.061 |
|  | Smoke |  | -0.217 | 5.908 | -17.626 | 6.879 | -0.91 | 0.373 |
|  | Drink |  | 0.397 | 5.303 | -2.397 | 19.597 | 1.62 | 0.119 |
|  | Bukuryoin |  | -0.063 | 6.234 | -14.277 | 11.580 | -0.22 | 0.831 |
|  | Heiisan |  | -0.265 | 7.594 | -21.882 | 9.615 | -0.81 | 0.428 |
| Total mood disturbance | BMI | 0.173 | -0.258 | 0.735 | -2.319 | 0.728 | -1.08 | 0.291 |
|  | Occupation |  | -0.034 | 4.780 | -10.648 | 9.180 | -0.15 | 0.879 |
|  | Marriage |  | -0.275 | 5.023 | -17.035 | 3.797 | -1.32 | 0.201 |
|  | Sleep |  | 0.166 | 5.294 | -7.269 | 14.691 | 0.70 | 0.491 |
|  | Appetite |  | -0.151 | 5.626 | -15.440 | 7.895 | -0.67 | 0.509 |
|  | Smoke |  | -0.006 | 6.655 | -13.947 | 13.657 | -0.02 | 0.983 |
|  | Drink |  | 0.021 | 5.973 | -11.935 | 12.840 | 0.08 | 0.940 |
|  | Bukuryoin |  | -0.088 | 7.022 | -16.463 | 12.662 | -0.27 | 0.789 |
|  | Heiisan |  | -0.091 | 8.554 | -19.874 | 15.605 | -0.25 | 0.805 |

*P<0.05

Only p > 0.05 result showed in this table.

POMS 2-A: Profile of Mood States Second Edition-Adult Short Form

**Supplementary Table 6. Paired t-test results of the Gastrointestinal Symptom Rating Scale (Supplementary).**

| **GSRS items** | **Mean ± SD** | | **Cohen’s d** | **95% CI** | | **t** | **P**  **value** |
| --- | --- | --- | --- | --- | --- | --- | --- |
|  | **Before** | **After** |  | **Lower Bound** | **Upper Bound** |  |  |
| Stomachache | 3.05 ± 1.96 | 2.41 ± 1.37 | -0.36 | -0.814 | 0.102 | 1.67 | 0.110 |
| Heartburn | 2.50 ± 1.65 | 2.18 ± 1.18 | -0.20 | -0.645 | 0.251 | 0.92 | 0.366 |
| Acid Reflux | 2.32 ± 1.64 | 1.91 ± 0.97 | -0.27 | -0.724 | 0.179 | 1.28 | 0.215 |
| Nausea | 2.55 ± 1.71 | 2.00 ± 1.35 | -0.40 | -0.859 | 0.063 | 1.87 | 0.076 |
| Rumbling | 2.23 ± 1.77 | 2.00 ± 1.20 | -0.16 | -0.607 | 0.285 | 0.76 | 0.459 |
| Burp | 2.00 ± 1.45 | 1.73 ± 1.32 | -0.43 | -0.897 | 0.032 | 2.03 | 0.056 |
| Fart | 2.77 ± 1.31 | 2.50 ± 1.41 | -0.22 | -0.669 | 0.229 | 1.03 | 0.315 |
| Diarrhea | 1.91 ± 1.34 | 1.73 ± 1.12 | -0.13 | -0.575 | 0.316 | 0.61 | 0.550 |
| Loose stools | 2.23 ± 1.31 | 1.91 ± 1.19 | -0.24 | -0.690 | 0.210 | 1.13 | 0.272 |
| Hard stools | 1.59 ± 1.33 | 1.59 ± 1.62 | 0.00 | -0.443 | 0.443 | 0.00 | 1.000 |
| Urgent need for defecation | 2.32 ± 1.70 | 2.14 ± 1.39 | -0.10 | -0.546 | 0.343 | 0.48 | 0.639 |
| Incomplete evacuation feeling | 2.09 ± 1.77 | 1.64 ± 0.95 | -0.33 | -0.788 | 0.124 | 1.56 | 0.135 |

*P<0.05

Only p > 0.05 result showed in this table.

GSRS:Gastrointestinal Symptom Rating Scale.

**Supplementary Table 7. Paired t test Result of Profile of Mood States Second Edition-Adult Short Form (Supplementary).**

| **POMS 2-A Items** | **Mean ± SD** | | **Cohen’s d** | **95% CI** | | **t** | **P**  **value** |
| --- | --- | --- | --- | --- | --- | --- | --- |
|  | **Before** | **After** |  | **Lower Bound** | **Upper Bound** |  |  |
| Friendliness | 49.91 ± 13.28 | 46.94 ± 13.78 | -0.30 | -0.674 | 0.064 | 1.72 | 0.095 |
| Tension | 52.91 ± 13.07 | 49.63 ± 10.38 | -0.26 | -0.626 | 0.107 | 1.47 | 0.152 |
| Fatigue | 53.66 ± 11.02 | 49.75 ± 10.05 | -0.35 | -0.719 | 0.024 | 1.97 | 0.058 |
| Vigor | 45.47 ± 10.16 | 45.06 ± 12.49 | -0.05 | -0.410 | 0.311 | 0.28 | 0.780 |
| Confusion | 54.50 ± 13.50 | 51.19 ± 11.31 | -0.25 | -0.617 | 0.116 | 1.42 | 0.167 |

*P<0.05

Only p > 0.05 result showed in this table.

POMS 2-A: Profile of Mood States Second Edition-Adult Short Form
